# Impact of homocysteine on acute ischemic stroke severity: possible role of aminothiols redox status

**DOI:** 10.1186/s12883-024-03681-5

**Published:** 2024-05-24

**Authors:** Wei-Chong Dong, Jia-Liang Guo, Lei Xu, Xin-Hui Jiang, Cheng-Hao Chang, Ye Jiang, Ying-Ze Zhang

**Affiliations:** 1 The School of Medicine, Nankai University, 94# Weijin Road, Tianjin, 300071 China; 2 Department of Pharmacy, The Second Hospital of Hebei Medical University, Shijiazhuang 050051, Hebei Province China; 3 Hebei Key Laboratory of Forensic Medicine, Shijiazhuang 050017, Hebei Province China; 4 Department of Orthopaedics, Hebei Medical University Third Hospital, 139# Ziqiang Road, Shijiazhuang 050000, Hebei Province China; 5 Department of Neurology, The Second Hospital of Hebei Medical University, Shijiazhuang 050051, Hebei Province China; 6 Department of Obstetrics and Gynecology, Aerospace Central Hospital, Beijing, 100049 China; 7 Rehabilitation Medicine Department of the Eighth Medical Center of PLA General Hospital, Beijing, 100091 China; 8 Department of Pharmaceutical Analysis, School of Pharmacy, Hebei Medical University, 361# East Zhongshan Road, Shijiazhuang 050017, Hebei Province China

**Keywords:** Homocysteine, Homocystine, Acute ischemic stroke, National Institutes of Health Stroke Scale, Aminothiols

## Abstract

**Background:**

Acute ischemic stroke (AIS) is one of the most common cerebrovascular diseases which accompanied by a disruption of aminothiols homeostasis. To explore the relationship of aminothiols with neurologic impairment severity, we investigated four aminothiols, homocysteine (Hcy), cysteine (Cys), cysteinylglycine (CG) and glutathione (GSH) in plasma and its influence on ischemic stroke severity in AIS patients.

**Methods:**

A total of 150 clinical samples from AIS patients were selected for our study. The concentrations of free reduced Hcy (Hcy), own oxidized Hcy (HHcy), free reduced Cys (Cys), own oxidized Cys (cysteine, Cyss), free reduced CG (CG) and free reduced GSH (GSH) were measured by our previously developed hollow fiber centrifugal ultrafiltration (HFCF-UF) method coupled with high performance liquid chromatography-tandem mass spectrometry (HPLC-MS/MS). The concentration ratio of Hcy to HHcy (Hcy/HHcy), Cys to Cyss (Cys/Cyss) were also calculated. The neurologic impairment severity of AIS was evaluated using National Institutes of Health Stroke Scale (NIHSS). The Spearman correlation coefficient and logistic regression analysis was used to estimate and perform the correlation between Hcy, HHcy, Cys, Cyss, CG, GSH, Hcy/HHcy, Cys/Cyss and total Hcy with NIHSS score.

**Results:**

The reduced Hcy and Hcy/HHcy was both negatively correlated with NIHSS score in AIS patients with *P* = 0.008, *r*=-0.215 and *P* = 0.002, *r*=-0.249, respectively. There was no significant correlation of Cys, CG, GSH, HHcy, Cyss, Cys/Cyss and total Hcy with NIHSS score in AIS patients with *P* value > 0.05.

**Conclusions:**

The reduced Hcy and Hcy/HHcy, not total Hcy concentration should be used to evaluate neurologic impairment severity of AIS patient.

## Introduction

Stroke stands as the second-leading contributor to mortality worldwide. With more than 12 million new cases reported in 2019, it represents 11.6% of all deaths. Simultaneously, it is the third-leading cause of combined death and disability, accounting for 5.7% of the total Disability-Adjusted Life Years (DALYs) [1, 2]. Since 2015, stroke has become the leading cause of death and disability in China, posing a significant threat to the health of its citizens as a major chronic non-communicable disease. With the accelerated aging of the population, the increasing prevalence of unhealthy life styles among residents, and the widespread exposure to stroke risk factors, the burden of stroke disease in China is on an explosive growth trend [3]. Especially, acute ischemic stroke (AIS) is the most common cerebrovascular disease, with its incidence increasing annually and mortality continuing to grow, seriously threatening people’s lives and health [4, 5]. Hyperhomocysteinemia is one of the most important risk factors for the development of AIS, along with diabetes, arterial hypertension, internal carotid artery stenosis, heart disease, lipid metabolism disorder and smoking [6].

Homocysteine (Hcy), a sulfur-containing amino acid with biological functions in methionine metabolism [7]. Elevated concentration of Hcy has been reported to be a risk factor shared by neurodegenerative conditions [8], cardiovascular disease [9], and vascular complication of diabetes [10]. Currently considered an independent risk factor for AIS, Hcy’s roles in atherosclerotic modification and plaque formation are associated with vascular endothelial injury and inflammation. However, the exact pathological mechanism associated with Hcy remains controversial [11, 12]. Clinical research shows that there are contradictory results in preventing primary and recurrent stroke and post-stroke rehabilitation by reducing Hcy level, and these results can not be satisfactorily explained at present [13, 14]. The effect of Hcy level on neurological and functional prognosis of AIS patients at admission is still unclear [6].

In those studies, a composite marker was used, so called ‘total Hcy’ referring to all redox form of Hcy, including reduced and oxidized or protein bound forms [15, 16]. In fact, homocysteine (Hcy) and its metabolically related aminothiols cysteine (Cys), cysteinylglycine (CG), and glutathione (GSH) contribute to the plasma redox thiol status, and play important roles in the pathogenesis of stroke [17]. These aminothiols exist in plasma as protein-bound and unbound forms (free forms) [18]. Since aminothiol fractions bounded with proteins are probably not biologically active, the free forms of aminothiol may be more likely to play a role in the pathogenesis of diseases than other forms of aminothiol [19]. The free fraction of the aminothiols include Hcy–Hcy disulfides or other mixed disulfides and thiols with a free sulfhydryl group (reduced forms). It has reported that approximately 70% of plasma total Hcy is bound to albumin, 30% is oxidized to disulfides to form homocysteine–homocysteine (Homocystine, HHcy) or homocysteine–cysteine, and only approximately 1% is present as free circulating Hcy [20]. Many literature examples showed that the presence of free reduced Hcy (Hcy) was significantly increased in the pathological state [21, 22]. It has also reported that free reduced Hcy (Hcy), rather than total Hcy (tHcy), were significantly elevated in patients with acute/subacute ischemic stroke [23].

Oxidative stress (OS) plays an important role in the pathogenesis of nervous tissue damage in the presence of stroke [24]. Aminothiols are part of the antioxidant system of plasma and are in a state of dynamic equilibrium between the disulphide and reduced forms [25, 26]. Their redox state may serve as a sensitive indicator of acute cerebrovascular insufficiency [6, 17]. The active involvement of thiols in oxidation processes gave reason to consider them as potential diagnostic and/or prognostic markers of ischemic stroke. Although various different oxidation stress indicators in stroke patients have been studied, clinical data on the plasma thiol homeostasis are insufficient and controversial [27]. Several studies have shown the relationship of total aminothiols with acute stroke, but the results were contradictory [6]. The low-molecular-weight aminothiol (Hcy, Cys, CG, GSH and others) are important components of the blood antioxidant defense system and are the most chemically active fractions of total aminothiols and can be used as markers of OS which are more sensitive than total aminothiols [17].

Some literature using the ratio of the reduced forms to the total content of each thiol to characterize the redox status [17, 27]. In fact, oxidative stress and redox signaling involve electron transfer reactions. As defined by the Nernst equation (*E*_h_ = *E*_0_ + *RT/nF* ln([oxidized]/ [reduced]) [28, 29]. The displacement from equilibrium of thiol/disulfide couples allows rapid and dynamic regulation, supports redox signaling, and represents a central target of nonradical mechanisms of oxidative stress [30]. Therefore, thiol/disulfide should be used to characterize the redox status more accurately than the ratio of the reduced forms to the total content of each thiol. However, there was no report about the relationship of stroke with the concentration ratio of free reduced to oxidized thiol in clinical patients. It also reported the reduced and oxidized Cys and GSH in rat brain tissues with cerebral ischaemia, but not about the concentration ratio of free reduced to own oxidized Hcy (Hcy/HHcy), and it also not studied in clinical patients [17]. In our previous work [31], we reported the concentration ratio of free reduced to own oxidized Hcy (Hcy/HHcy) was a more accurate biomarker to evaluate oxidative stress in rat plasma with osteoporosis. But it has not been validated in clinic and the relationship between Hcy/HHcy and stroke remains unclear.

Therefore, in present work, we aimed to explore the relationship of aminothiols concentration and the concentration ratio of free reduced to their own oxidized aminothiols with neurologic impairment severity of AIS (evaluated using National Institutes of Health Stroke Scale [NIHSS]) in clinical AIS patients.

## Materials and methods

### Materials and instruments

The hollow fiber was purchased from Hebei Heping Medical Equipment Factory (shijiazhuang, China). The molecular cut-off was 10 kDa. The wall thickness of this fiber was 150 μm and the inner diameter was 1000 μm. The slim glass tubes were 7 cm of height and 4.5 mm of inner diameter. A LC-20AD high-performance liquid chromatography (Shimadzu, Japan) system coupled to an API 4000^+^ (AB SCIEX, Los Angeles, CA, USA) Triple quadrupole mass spectrometer equipped were used for chromatographic analysis.

### Sample collection and preparation

**Fig. 1:**
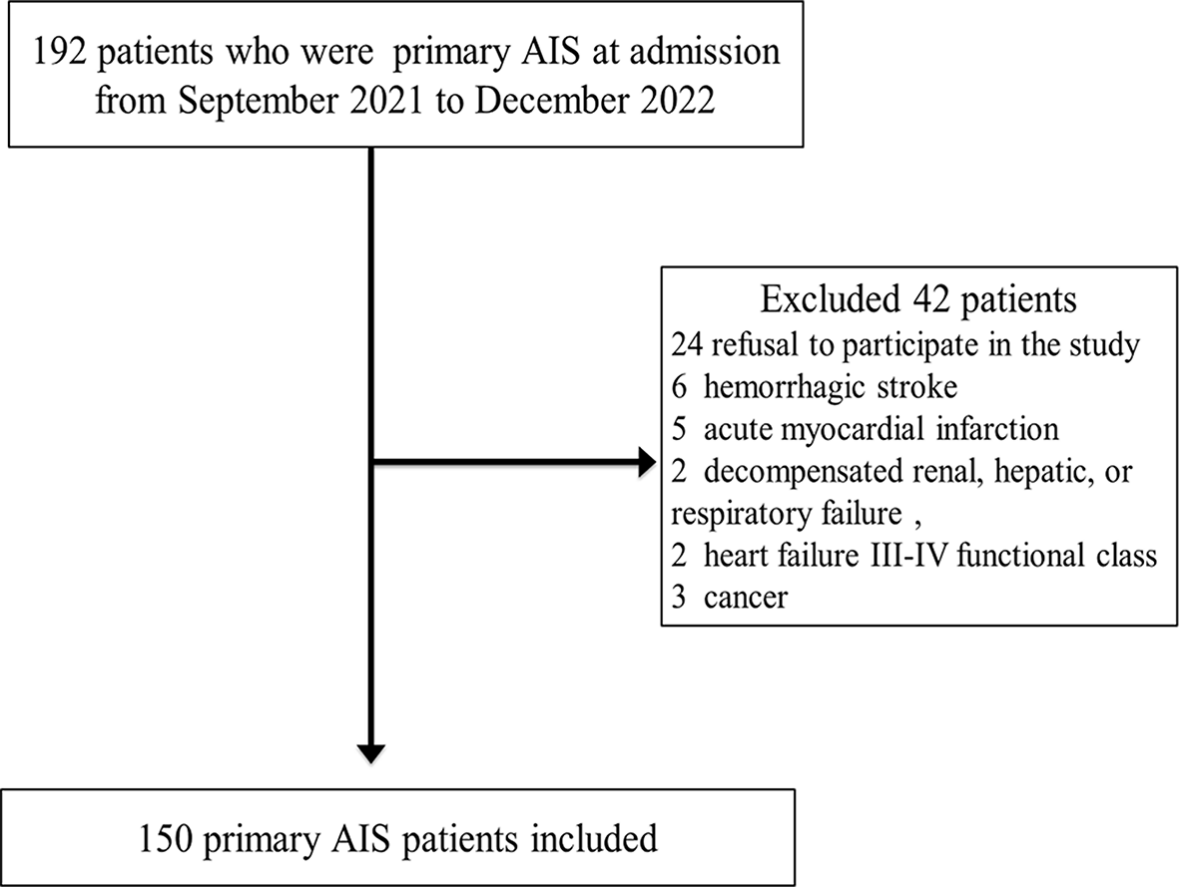
The flow chart of patient inclusion

The study protocol was approved by the Ethics Committee of The Hebei Medical University (No.2021026). A total of 150 patients diagnosed with AIS in the Second Hospital of Hebei Medical University were selected for our study from September 2021 to December 2022 and all subjects or their guardians provided informed consent. A flow chart of patient inclusion was show in Fig. 1. The criteria for exclusion from the study were refusal to participate in the study, hemorrhagic stroke, acute myocardial infarction, decompensated renal, hepatic, or respiratory failure, heart failure III-IV functional class, and cancer. Information on hypertension, diabetes mellitus, and heart disease was based on the medical history and clinical data. The NIHSS score were evaluated jointly by the residents of the Neurology Department or physicians with higher professional titles. The Alcohol drinkers were determined as individuals who drank > 1 standard drink (10 g alcohol) per week for > 2 years. Smokers were defined as individuals who smoked > 5 cigarettes per day for at least 2 years. The subtype of IS was determined according to the TOAST (Trial of ORG 10,172 in Acute Stroke Treatment) classification criteria (1 large artery atherosclerosis; 2 cardioembolism; 3 small vessel occlusion; 4 Other determined causes, e.g., dissection, mitochondrial or genetic; 5 strokes of unknown causes).

**Fig. 2:**
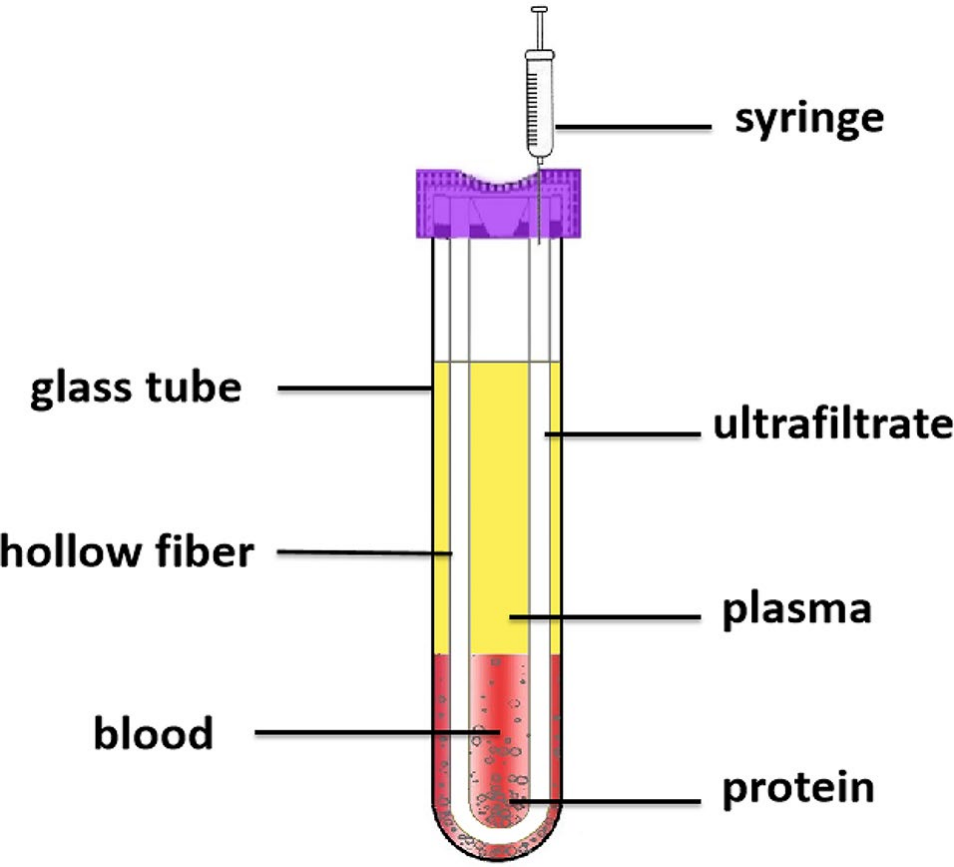
Hollow fiber centrifugal ultrafiltration (HFCF-UF) device

After admission, about 2mL blood were collected to EDTA anticoagulant tube and centrifuged for 5 min at 2.4 × 10^3^ g for determination of total Hcy concentration with enzymatic cycling methods. Another 1mL blood sample were prepared according to our previously developed hollow fiber centrifugal ultrafiltration (HFCF-UF) method, as it show in Fig. 2. The concentrations of free reduced Hcy (Hcy), own oxidized Hcy (HHcy), free reduced Cys (Cys), own oxidized Cys (cysteine, Cyss), CG and GSH were measured by HPLC-MS/MS and the concentration ratio of Hcy to HHcy (Hcy/HHcy), Cys to Cyss (Cys/Cyss) were also calculated [31, 32].

### Statistical analysis

SPSS 24.0 software was used to perform a statistical analysis. The quantitative data (or measurement data) was described by mean ± standard deviation (SD). The Spearman correlation coefficient was used to estimate the correlation between total Hcy concentration and the Hcy/HHcy with NIHSS score and the data assessments were double tailed. A logistic regression analysis was performed to correlate the Hcy/HHcy and total Hcy concentration with NIHSS score. The values of *P* < 0.05 were considered to be statistically significant.

## Results

### Result of clinical patients date

A total of 150 clinical plasma samples from AIS patients were collected. The sample information and corresponding concentration results of Hcy, HHcy, Cys, Cyss, CG, GSH are presented in Table 1.

**Table 1:** The characteristics of the 150 plasma samples from AIS subject patients

Characteristic	mean ± SD, [range]
Gender (male/female)	109/41
Age (years)	61.4 ± 10.9[25–85]
Hypertension, *n* (%)	122 (81.3%)
Diabetes mellitus, *n* (%)	66 (44.0%)
Heart disease, *n* (%)	46 (30.7%)
Drinking, *n* (%)	71 (47.3%)
Smoking, *n* (%)	67 (44.7%)
Medication usage	
Aspirin, *n* (%)	87 (58.0%)
Statins, *n* (%)	141 (94.0%)
Clopidogrel, *n* (%)	65 (43.3%)
Stroke subtype	
Large artery atherosclerosis, *n* (%)	16 (10.7%)
Cardioembolism, *n* (%)	41 (27.3%)
Small vessel occlusion, *n* (%)	50 (33.3%)
Other determined causes, *n* (%)	15 (10.0%)
strokes of unknown causes, *n* (%)	28 (18.7%)
Hyperhomocysteinemia, *n* (%)	24 (16.0%)
NIHSS at admission	6 [0–25]
Total Hcy concentration (µmol·L^− 1^)	16.49 ± 9.19 [4.29–62.08]
Reduced Hcy concentration (Hcy, µmol·L^− 1^)	0.65 ± 0.64 [0.06–3.37]
Reduced Cys concentration (Cys, µmol·L^− 1^)	9.88 ± 7.68[0.44–34.20]
Reduced CG concentration (CG, µmol·L^− 1^)	58.32 ± 68.01[0.36–651]
Reduced GSH concentration (GSH, µmol·L^− 1^)	28.25 ± 61.45[0.55–471]
Own oxidized Hcy concentration (HHcy, µmol·L^− 1^)	0.0168 ± 0.0123 [0.0041–0.0704]
Own oxidized Cys concentration (Cyss, µmol·L^− 1^)	3.76 ± 2.86 [0.0142-15.50]
The concentration ratio of Hcy/HHcy	42.72 ± 37.85 [1.78-207.38]
The concentration ratio of Cys/Cyss	4.66 ± 7.24 [0.184–46.13]

### Correlation of hcy with NIHSS

**Fig. 3:**
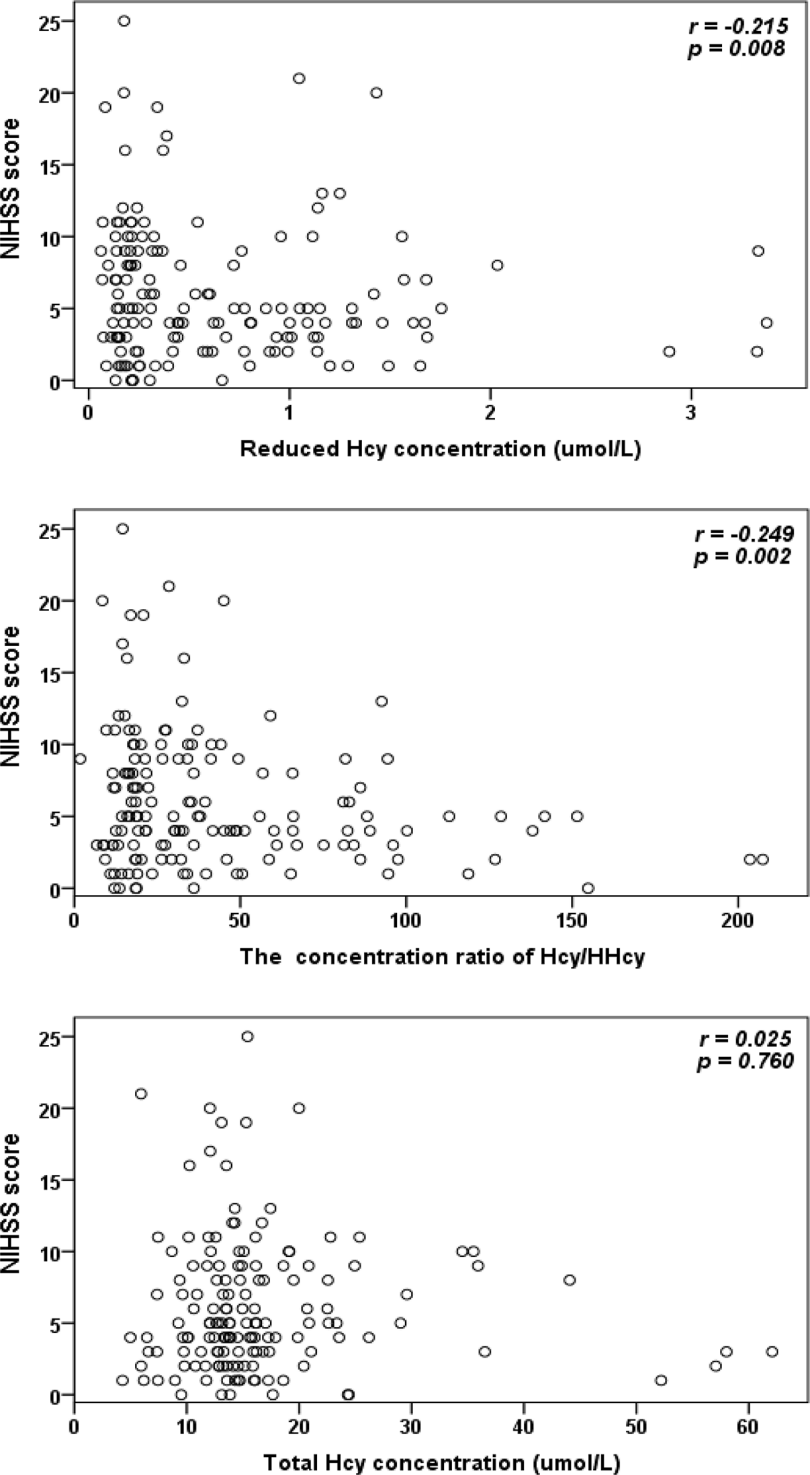
The results of the Spearman’s rank correlation for reduced Hcy, Hcy/HHcy and Total Hcy concentration with NIHSS score

The results of the Spearman’s rank correlation for Hcy, HHcy, Cys, Cyss, CG, GSH, Hcy/HHcy, Cys/Cyss and total Hcy with NIHSS score are shown in Table 2. The reduced Hcy concentration (Hcy) and Hcy/HHcy were both negatively correlated with NIHSS score, and total Hcy was not correlated with NIHSS score in AIS patients, as it shown in Fig. 3. There was no correlation of Cys, CG, GSH, HHcy, Cyss, and Cys/Cyss with NIHSS score in AIS patients.

**Table 2:** The results of Spearman’s Correlation Analysis Between Hcy concentration with NIHSS score

Item	Variables	Correlation coefficient, *r*	*P*
NIHSS	Reduced Hcy (Hcy)	-0.215	0.008*
	Reduced Cys (Cys)	-0.096	0.243
	Reduced CG (CG)	-0.042	0.611
	Reduced GSH (GSH)	-0.063	0.445
	Own oxidized Hcy (HHcy)	0.013	0.877
	Own oxidized Cys (Cyss)	-0.086	0.297
	Hcy/HHcy	-0.249	0.002*
	Cys/Cyss	0.005	0.954
	Total Hcy	0.025	0.760

* Correlation is significant at the 0.01 level (2-tailed)

### Logistic regression analysis

Refer to Table 3 for the results of logistic regression analysis. Binary logistic regression model was performed to estimate the odds ratios (ORs) and 95% confidence intervals (CIs) of Hcy, Cys, CG, GSH, Hcy/HHcy, Cys/Cyss and total Hcy for neurologic impairment severity with the NIHSS score as the dependent variable and categorized into two groups (< 7 or ≥ 7). Age, Gender, Hypertension, Diabetes mellitus, Heart disease, Drinking, Smoking, Medication usage (Aspirin, Statins, Clopidogrel), Stroke subtype and Hyperhomocysteinemia were also adjusted in the full adjustment model. Reduced Hcy concentration was associated with neurologic impairment severity in AIS patients when above variables were adjusted. The concentration ratio of HHcy/Hcy were still associated with neurologic impairment severity in AIS patients with and without correction. There were no significant association of Cys, CG, GSH, Cys/Cyss and total Hcy with NIHSS score with the value of *P* > 0.05.

**Table 3:** The results of logistic regression analysis of Hcy, Cys, CG, GSH, Hcy/HHcy, Cys/Cyss and total Hcy for NIHSS score

Model	Variables	OR	95% CI	*P*
Model 1	reduced Hcy	0.576	0.311–1.067	0.080
	reduced Cys	0.978	0.935–1.022	0.318
	reduced CG	0.998	0.992–1.004	0.487
	reduced GSH	0.995	0.988–1.003	0.235
	Total Hcy	1.001	0.965–1.038	0.956
	Hcy/HHcy	0.980	0.967–0.993	0.003**
	Cys/Cyss	1.015	0.971–1.062	0.503
Model 2	reduced Hcy	0.515	0.273–0.971	0.040*
	reduced Cys	0.977	0.934–1.022	0.306
	reduced CG	0.998	0.992–1.003	0.424
	reduced GSH	0.995	0.987–1.003	0.186
	Total Hcy	1.000	0.964–1.038	0.996
	Hcy/HHcy	0.978	0.965–0.992	0.001**
	Cys/Cyss	1.015	0.970–1.061	0.528
Model 3	reduced Hcy	0.520	0.277–0.978	0.042*
	reduced Cys	0.981	0.937–1.027	0.407
	reduced CG	0.998	0.992–1.004	0.575
	reduced GSH	0.995	0.987–1.004	0.290
	Total Hcy	0.997	0.961–1.035	0.875
	Hcy/HHcy	0.980	0.966–0.993	0.004**
	Cys/Cyss	1.013	0.968–1.061	0.581
Model 4	reduced Hcy	0.508	0.271–0.950	0.034*
	reduced Cys	0.995	0.948–1.045	0.843
	reduced CG	0.999	0.993–1.005	0.723
	reduced GSH	0.995	0.986–1.004	0.287
	Total Hcy	1.000	0.962–1.040	0.980
	Hcy/HHcy	0.980	0.966–0.995	0.007**
	Cys/Cyss	1.009	0.959–1.062	0.723

Model 1: unadjusted; Model 2: adjusted by age + sex; Model 3: adjusted by age + sex + Hypertension + Diabetes mellitus + Heart disease; Model 4: adjusted by age + sex + Hypertension + Diabetes mellitus + Heart disease + drinking + smoking + medication usage (Aspirin + Statins + clopidogrel) + stroke subtype + hyperhomocysteinemia. * *P* < 0.05; ** *P* < 0.01

## Discussions

### The relationship of hcy with neurological deficits

Multiple studies since the end of the twentieth century showed that the risk of ischemic stroke depends on total Hcy levels [33]. A number of studies have shown a positive association of the total Hcy level with the neurological deficit at admission and 7 days after ischemic stroke [34, 35]. It has also reported that the total Hcy were correlated with the NIHSS score after stent treatment in patients with AIS and total Hcy was one of the factors related to the NIHSS score changes after the cerebrovascular stent implantation [36]. Conversely, some publications showed no significant prognostic effect of tHcy on neurological deficits. Li et al. [37] reported the clinical contribution of tHcy to the functional IS outcome in the first 3 months was negligible, despite the reliable association of these indicators.

In our present work, Fig. 3 shows that total Hcy concentration was not significantly correlated with NIHSS score at admission in AIS patients (*P* = 0.760, *r*=-0.025). The logistic regression analysis in Table 3 also shows the total Hcy concentration was not significantly correlate with NIHSS score in AIS patients (*P* = 0.956), when age, sex, hypertension, diabetes mellitus, heart disease, drinking, smoking, medication usage (Aspirin, Statins, Clopidogrel), stroke subtype and hyperhomocysteinemia were corrected, the association was still not significant (*P* = 0.980). However, there was no significantly association between free reduce Hcy and NIHSS score without correction (*P* = 0.080). When age, sex, hypertension, diabetes mellitus, heart disease, drinking, smoking, medication usage (Aspirin, Statins, clopidogrel), stroke subtype and Hyperhomocysteinemia were corrected, the association of free reduce Hcy and NIHSS score was significant (*P* = 0.040, *P* = 0.042 and *P* = 0.034). The results were consistent with those reported in the literature. Since it has concluded that reduced Hcy was the deleterious form of homocysteine [21, 22] and reduced Hcy (Hcy), rather than total Hcy (tHcy), were significantly correlate in patients with acute/subacute ischemic stroke [23, 27].

It was found previously on acute cerebral ischaemia models in rats that the reduced forms of aminothiols in blood plasma undergo a significant drop [17, 38]. Our results showed that reduced forms of Hcy decreased significantly with the increase of NIHSS at admission which were in consistent with the literature. There was also reported that Cys and CG reduced forms undergo a significant drop during the first week after stroke in patients. At the same time, reduced forms of GSH and Hcy do not change significantly [27]. In our present work, there was no significant correlation of Cys, CG, GSH with NIHSS score in AIS patients. It has reported that the plasma aminothiols are actively involved in oxidation reactions at early stages of cerebral ischaemia; therefore, their redox state may serve as a sensitive indicator of acute cerebrovascular insufficiency [6, 17]. Since the fraction of reduced Hcy is the most chemically active among the other aminothiols forms. The decrease of reduced Hcy in AIS indicates amplification of oxidative processes and insufficiency of antioxidant systems involved in the reduction of thiols [27]. The different results also can be explained by a number of factors and individual properties influencing aminothiols metabolism. Such factors and more standardized clinical trials are in need of further studying.

### The relationship of Hcy/HHcy and Cys/Cyss with neurological deficits

Aminothiols are important components of the blood antioxidant defense system. They are in a state of dynamic equilibrium between disulfide and reduced forms [25, 26]. It reported the ratio of the reduced forms to the total content of each thiol can be used to characterize the redox status (RS) [39]. But according to Nernst equation, oxidative stress and redox signaling involve electron transfer reactions, and *E*_h_ values provide convenient parameters to describe relationships between various biochemicals undergoing oxidation-reduction reactions. *E*_h_ is dependent upon both the inherent properties of the chemical to accept or donate electrons, expressed in the standard potential (*E*_0_), and the concentrations of the acceptor (oxidized) and donor (reduced) species of the couple [29]. Thiol/disulfide couples have diverse functions in cell signaling, protein regulation, and macromolecular structure and trafficking. Thiol/disulfide as redox buffers to maintain redox homeostasis and resist or facilitate oxidation of protein thiols to change protein functions and transduce signals [40]. This displacement from equilibrium allows rapid and dynamic regulation, supports redox signaling, and represents a central target of nonradical mechanisms of oxidative stress [41]. Therefore, thiol/disulfide should be used to characterize the redox status more accurately. A number of studies have reported the relationship of total Hcy or reduced Hcy level with the neurological deficit in stroke patients [27, 33, 37]. But there has no report about the relationship of Hcy/HHcy with neurologic impairment severity in stroke.

In our present work, Fig. 3; Table 2 shows that Hcy/HHcy was negatively correlated with NIHSS in AIS patients (*P* = 0.002, *r*=-0.249). The logistic regression analysis in Table 3 also shows the Hcy/HHcy was significantly correlate with NIHSS score in AIS patients (*P* = 0.003), when age, sex, hypertension, diabetes mellitus, heart disease, drinking, smoking, medication usage (Aspirin, Statins, Clopidogrel), stroke subtype and hyperhomocysteinemia were corrected, the association was still significant (*P* = 0.001, *P* = 0.004 and *P* = 0.007). It maybe explained the value of Hcy/HHcy decreases with the increase of NIHSS in stroke when redox homeostasis was disrupted. The reduced forms of homocysteine (Hcy) decrease, and oxidized forms (HHcy) increase with oxidative stress occurs, which induce the decreases of the ratio (Hcy/HHcy). It was accord with our previously report in rat plasma with osteoporosis [31]. Although the increase of the HHcy were not significantly correlated with NIHSS increase in AIS patients in present work, it maybe the due the HHcy concentration was very low and significant individual differences. Ivanov et al. [17] have reported the reduced and oxidized Cys and GSH in rat brain tissues with cerebral ischaemia, but not about the Hcy/HHcy, due to the level of Hcy, especially the oxidized Hcy (HHcy) in the brain was too low to allow quantitative determination.

Anther thiol/disulfide couples, cysteine/cystine (Cys/Cyss) is exploited for a large number of biological processes [28]. The reduction potentials (*E*_h_) for the Cys/CySS in plasma are useful indicators of systemic oxidative stress and other medically relevant physiological states. Such as, plasma E_h_(Cys/Cyss) was found to be more oxidized in adults chronically exposed to arsenic [42], adults acutely exposed to acetaminophen [43], and in children with autism [44]. While, there has no report about the relationship of Cys/Cyss with neurologic impairment severity in stroke.It has reported that the concentration ratio of Cys/Cyss in rat brain tissues with cerebral ischaemia were not changed significantly [17]. In our present work, the Cys/Cyss was not significantly correlate with NIHSS score in AIS patients (*P* = 0.503), when age, sex, hypertension, diabetes mellitus, heart disease, drinking, smoking, medication usage (Aspirin, Statins, Clopidogrel), stroke subtype and hyperhomocysteinemia were corrected, the association was still not significant (*P* = 0.723). We have previously reported that the Hcy/HHcy and Cys/Cyss could be suitable biomarkers for oxidative stress and especially Hcy/HHcy is 12 times more sensitive than Cys/Cyss in osteoporosis [31]. Therefore, Hcy/HHcy can be considered as a potential diagnostic and/or prognostic markers of neurologic impairment severity in AIS.

### Practical implications and feasibility of measuring Hcy/HHcy

It has also reported a redox imbalance in AIS patients and an antioxidant-based therapy could be beneficial for the recovery [27]. OS markers and the status of various antioxidant systems in organisms can be used as indicators to diagnose and predict the development of stroke. In clinical practice, determination of Hcy/HHcy only needs one-step blood sampling and test, which can be realized in the clinical routine laboratory. The method is simple and rapid, and is suitable for routine analysis of clinical samples [31, 32]. Importantly, the results can indicate the oxidative stress state of the body and may indicate the severity of neurological deficit, and can also be of diagnostic importance both at an early stage of ischemic stroke development and during its monitoring.

### Limitations

In our present work, there are following limitations which should be focus on in further study:

Firstly, the initial purpose of our research was to determine the total, reduced and oxidized forms of Hcy, Cys, CG and GSH simultaneously, but in our present work, we only measured total Hcy, reduced Hcy (Hcy) and oxidized Hcy (HHcy), reduced Cys (Cys) and oxidized Cys (Cyss), reduced CG and reduced GSH. Since our developed method could not realize to measure oxidized CG and oxidized GSH (GSSG), total Cys, total CG and total GSH [31, 32]. So another pair of thiol/disulfide couples, GSH/GSSG [29, 30], which is a very important indicators of oxidative stress and disease risk were not studied. But since the Hcy was usually used as a biomarker in clinic and most related to stroke, so we mainly studied the total Hcy, reduced Hcy (Hcy), and oxidized Hcy (HHcy) in present work. So further study would also focus on improving analysis method and explore the relationship GSH/GSSG or total aminothiols with neurologic impairment severity in AIS in further study.

Secondly, unfortunately, only one-time point (Immediately after admission) was collected in present work. Because different patients have different treatment methods and schemes, their recovery status is also different, so we did not collect blood samples at subsequent unified time points. Some patients measured total Hcy during the follow-up treatment or before discharge, but some patients did not measure it afterwards. So we did not collect blood samples at subsequent unified time points. Therefore, we regret that we can not study the Hcy level change over the course of follow-up and the influence of treatment, such as IV-tPA, endovascular thrombectomy or reperfused after thrombectomy in present work. We would focus on the influence of treatment on homocystein in our further study.

Thirdly, the sample size in our present study was small, and the samples come from clinical patients with all sorts of complicating factors and do not have normal controls who were without any risk of stroke. It was only a preliminary finding in our present work and we hope it can arouse the interest of researchers. A rigorous multi-center clinical trial with large sample size would be conducted to validate the results in the further study.

Lastly, in present work, Age, Gender, Hypertension, Diabetes mellitus, Heart disease, Drinking, Smoking, Medication usage, Stroke subtype and Hyperhomocysteinemia were adjusted in logistic regression analysis. There are also some other potential impact of confounding factors should be considered, such as time from stroke onset to blood sampling. Since the time from stroke onset to blood sampling were not all collected accurately. So we did not added it as a variable to adjusted. But we would explore this factor in our further study.

## Conclusions

In the present work, we explored the relationship of four aminothiols (Hcy, Cys, CG, GSH) with neurologic impairment severity of AIS patient. The reduced Hcy and Hcy/HHcy was negatively correlated with NIHSS score in AIS patients. There was no significant correlation of Cys, CG, GSH, HHcy, Cyss, Cys/Cyss and total Hcy with NIHSS score in AIS patients. The value of Hcy/HHcy decreases with severe neurologic impairment severity in AIS patient when redox homeostasis was disrupted inducing the reduced forms of homocysteine (Hcy) decrease. The reduced Hcy and Hcy/HHcy, not total Hcy concentration should be used to evaluate neurologic impairment severity of AIS patient.

## Data Availability

The authors confirm that all data generated or analyzed during this study are included in this published article.

## References

[1] Feigin VL, Stark BA, Johnson CO (2021). Global, regional, and national burden of stroke and its risk factors, 1990–2019: a systematic analysis for the global burden of Disease Study 2019. Lancet Neurol.

[2] Tu W, Wang D, Yan F (2023). China stroke surveillance report 2021. Mil Med Res.

[3] Briones-Valdivieso C, Briones F, Orellana-Urzúa S (2024). Novel Multi-antioxidant Approach for ischemic stroke therapy targeting the role of oxidative stress. Biomedicines.

[4] Towfighi A, Saver JL (2011). Stroke declines from third to fourth leading cause of death in the United States: historical perspective and challenges ahead. Stroke.

[5] Gorelick PB (2019). The global burden of stroke: persistent and disabling. Lancet Neurol.

[6] Maksimova MY, Ivanov AV, Virus ED (2021). Impact of glutathione on acute ischemic stroke severity and outcome: possible role of aminothiols redox status. Redox Rep.

[7] Stipanuk MH (2004). Sulfur amino acid metabolism: pathways for production and removal of homocysteine and cysteine. Annu Rev Nutr.

[8] Haan MN, Miller JW, Aiello AE (2007). Homocysteine, B vitamins, and the incidence of dementia and cognitive impairment: results from the Sacramento Area latino study on aging. Am J Clin Nutr.

[9] Wald DS, Law M, Morris JK (2002). Homocysteine and cardiovascular disease: evidence on causality from a meta-analysis. B M J.

[10] Muzurović E, Kraljević I, Solak M (2021). Mikhailidis. Homocysteine and diabetes: role in macrovascular and microvascular complications. J Diabetes Complications.

[11] Sibrian-Vazquez M, Escobedo JO, Lim S (2010). Homocystamides promote free-radical and oxidative damage to proteins. Proc Natl Acad Sci U S A.

[12] McCully KS (2015). Homocysteine and the pathogenesis of atherosclerosis. Expert Rev Clin Pharmacol.

[13] Spence JD (2016). Homocysteine lowering for stroke prevention: unravelling the complexity of the evidence. Int J Stroke.

[14] Martí-Carvajal AJ, Solà I, Lathyris D (2017). Homocysteine-lowering interventions for preventing cardiovascular events. Cochrane Database Syst Rev.

[15] Zinellu A, Sotgia S, Scanu B (2010). Determination of homocysteine thiolactone, reduced homocysteine, homocystine, homocysteine–cysteine mixed disulfide, cysteine and cystine in a reaction mixture by over imposed pressure/voltage capillary electrophoresis. Talanta.

[16] Ivanov AV, Luzyanin BP, Kubatiev AA (2012). The Use of N-Ethylmaleimide for Mass Spectrometric detection of homocysteine fractions in blood plasma. Bull Exp Biol Med.

[17] Ivanov AV, Alexandrin VV, Paltsyn AA (2017). Plasma low-molecular-weight thiol/disulphide homeostasis as an early indicator of global and focal cerebral ischaemia. Redox Rep.

[18] Williams RH, Maggiore JA, Reynolds RD (2001). Novel approach for the determination of the redox status of homocysteine and other aminothiols in plasma from healthy subjects and patients with ischemic stroke. Clin Chem.

[19] Ma L, He J, Zhang XQ (2014). Determination of total, free, and reduced homocysteine and related aminothiols in uremic patients undergoing hemodialysis by precolumn derivatization HPLC with fluorescence detection. RSC Adv.

[20] Espina JG, Montes-Bayón M, Blanco-González E, Sanz-Medel A (2015). Determination of reduced homocysteine in human serum by elemental labelling and liquid chromatography with ICP-MS and ESI-MS detection. Anal Bioanal Chem.

[21] Sj¨oberg B, Anderstam B, Suliman M (2006). Plasma reduced homocysteine and other aminothiol concentrations in patients with CKD. Am J Kidney Dis.

[22] Chambers JC, Ueland PM, Wright M (2001). Investigation of relationship between reduced, oxidized, and protein-bound homocysteine and vascular endothelial function in healthy human subjects. Circ Res.

[23] Yang TH, Chang CY, Hu ML (2004). Various forms of homocysteine and oxidative status in the plasma of ischemic-stroke patients as compared to healthy controls. Clin Biochem.

[24] Valko M, Leibfritz D, Moncol J (2007). Free radicals and antioxidants in normal physiological functions and human disease. Int J Biochem Cell Biol.

[25] Escobar J, Sánchez-Illana Á, Kuligowski J (2016). Development of a reliable method based on ultra-performance liquid chromatography coupled to tandem mass spectrometry to measure thiol-associated oxidative stress in whole blood samples. J Pharm Biomed Anal.

[26] Tomaiuolo M, Vecchione G, Grandone E (2006). A new method for determination of plasma homocystine by isotope dilution and electrospray tandem mass spectrometry. J Chromatogr B Analyt Technol Biomed Life Sci.

[27] Maksimova MY, Ivanovb AV, Virusb ED (2019). Disturbance of thiol/disulfide aminothiols homeostasis in patients with acute ischemic stroke stroke: preliminary findings. Clin Neurol Neurosurg.

[28] Zheng YX, Ritzenthaler JD, Burke TJ (2018). Age-dependent oxidation of extracellular cysteine/cystine redox state (eh (Cys/CySS)) in mouse lung fibroblasts is mediated by a decline in Slc7a11 expression. Free Radic Biol Med.

[29] Jones DP, Liang YL (2009). Measuring the poise of thiol/disulfide couples in vivo. Free Radic Biol Med.

[30] Watson WH, Ritzenthaler JD, Peyrani P (2019). Plasma cysteine/cystine and glutathione/glutathione disulfide redox potentials in HIV and COPD patients. Free Radic Biol Med.

[31] Dong WC, Guo JL, Jiang XH (2023). A more accurate indicator to evaluate oxidative stress in rat plasma with osteoporosis. RSC Adv.

[32] Dong WC, Guo JL, Zhao MQ (2021). A simple and accurate HFCF-UF method for the analysis of homocysteine, cysteine, cysteinyl-glycine, and glutathione in human blood. Anal Bioanal Chem.

[33] Wu X, Zhou Q, Chen Q (2020). Association of homocysteine level with risk of stroke: a dose-response meta-analysis of prospective cohort studies. Nutr Metab Cardiovasc Dis.

[34] Harris S, Rasyid A, Kurniawan M (2019). Association of high blood homocysteine and risk of increased severity of ischemic stroke events. Int J Angiol.

[35] Yao ES, Tang Y, Xie MJ (2016). Elevated homocysteine level related to poor outcome after thrombolysis in acute ischemic stroke. Med Sci Monit.

[36] Chen Q, Ling WT, Han DK (2022). Study on the Association of Homocysteine and C-Reactive protein with neurofunctional changes in patients with Acute Ischemic Stroke after Endovascular Stent Treatment. Neuropsychiatr Dis Treat.

[37] Li L, Ma X, Zeng L (2020). Impact of homocysteine levels on clinical outcome in patients with acute ischemic stroke receiving intravenous thrombolysis therapy. PeerJ.

[38] Ivanov AV, Alexandrin VV, Paltsyn AA (2018). Metoprolol and nebivolol prevent the decline of the redox status of low-molecular-weight aminothiols in blood plasma of rats during acute cerebral ischemia. J Cardiovasc Pharmacol.

[39] Mansoor MA, Svardal AM, Schneede J (1992). Dynamic relation between reduced, oxidized, and protein-bound homocysteine and other thiol components in plasma during methionine loading in healthy men. Clin Chem.

[40] Kemp M, Go YM, Jones DP (2008). Nonequilibrium thermodynamics of thiol/disulfide redox systems: a perspective on redox systems biology. Free Radic Biol Med.

[41] Jones DP (2008). Radical-free biology of oxidative stress. Am J Physiol Cell Physiol.

[42] Hall MN, Niedzwiecki M, Liu X (2013). Chronic arsenic exposure and blood glutathione and glutathione disulfide concentrations in Bangladeshi adults. Environ Health Perspect.

[43] Mannery YO, Ziegler TR, Park Y (2010). Oxidation of plasma cysteine/cystine and GSH/GSSG redox potentials by acetaminophen and sulfur amino acid insufficiency in humans. J Pharmacol Exp Ther.

[44] Rose S, Melnyk S, Trusty TA et al. Intracellular and extracellular redox status and free radical generation in primary immune cells from children with autism. *Autism Res Treat*. 2012; 2012:986519. 10.1155/2012/986519 PMC3420377 22928106

